# Complete genome sequence of *Colletotrichum jinshuiense*, the causal agent of goldthread anthracnose disease

**DOI:** 10.1128/mra.00380-24

**Published:** 2025-01-27

**Authors:** Zuo-Qian Wang, Shu Zhang, Xiang-Qian Chang, Xiao-Lin Yang, Jing-Mao You, Yang Zhou, Chao-Xi Luo, Liang Lv

**Affiliations:** 1 Institute of Plant Protection and Soil Fertilizer, Hubei Academy of Agricultural Sciences, Wuhan, China; 2 Hubei Key Laboratory for Crop Diseases, Insect Pests and Weeds Control, Key Laboratory of Integrated Pest Management on Crops in Central China, Ministry of Agriculture, Wuhan, China; 3 Institute of Chinese Herbal Medicines, Hubei Academy of Agricultural Sciences, Enshi, China; 4 Oil Crops Research Institute, Chinese Academy of Agricultural Sciences, Wuhan, China; 5 Department of Plant Pathology, College of Plant Science and Technology, Huazhong Agricultural University, Wuhan, China; University of California Riverside, Riverside, California, USA

**Keywords:** genome sequence, *Colletotrichum jinshuiense*, Pacific Biosciences, anthracnose disease

## Abstract

The complete genome sequence of *Colletotrichum jinshuiense*, a goldthread anthracnose pathogen, was sequenced using PacBio Revio and MGI DNBSEQ-T7 PE150. It contains 10 chromosomes, 5 mini chromosomes, a circular mitochondrial chromosome, and 13,129 genes predicted with RNA-Seq data in a 52.13-Mb genome with an *N*_50_ of 5.05 Mb.

## ANNOUNCEMENT

*Colletotrichum*, the causal agent of plant anthracnose diseases, is among the most pathogenic fungi, with some species also affecting humans and animals (1–4). It contains 16 species complexes (5), including *Colletotrichum jinshuiense* within the *Colletotrichum dematium* species complex, which has been found to infect pears and persimmons (6, 7). In our previous study, the pathogen EsH8 was single-spore isolated from goldthread leaves in a field in Enshi, Hubei, China, and deposited in the China Centre for Type Culture Collection (collection-number AF 2024014, public depository). It was identified as *C. jinshuiense* through multi-gene phylogenetic analysis (8). However, the *C. jinshuiense* genome remains unpublished.

In this study, we report a complete genome assembly of EsH8 using third and second-generation sequencing technologies, annotated with RNA-seq data. Mycelia were cultured in potato dextrose broth (PDB) for 2 days, and genomic DNA was extracted using the DNeasy Plant Kit (Qiagen, Germany) (9). DNA was sheared using g-TUBEs (Covaris, USA), enriched, and purified with magnetic beads, followed by damage repair and end repair using SMARTbell Express Template Prep Kit 2.0 (PacBio, USA). Fragments were then selected and purified using a BluePippin system (Sage Science, USA). The library was built with the PacBio Binding Kit (PacBio, USA). The product was purified by AMPure PB Beads (PacBio, USA) and sequenced on a PacBio Revio sequencer (PacBio, USA) to attain a 192.37× coverage depth. Read adapter trimming and low-quality region filtering based on the signal-to-noise ratio were performed using Smrtlink with high-quality region finder (PacBio, USA). After *de novo* genome assembly using Hifiasm (version 0.19) (10), 15 scaffolds and 1 mitochondrial sequence were constructed, and *N*_50_ was 5.05 Mb with a total genome size of 52.13 Mb. All 15 scaffolds contain telomere identical repeats at the end, which were analyzed using biotool (11). Ten of them were identified as chromosomes, and the remaining five short scaffolds were identified as mini chromosomes (12). Second-generation sequences were sequenced by MGI DNBSEQ-T7 PE150 (MGI Tech Co., Ltd, China) with 94.71× sequencing depth. Completeness of the assembly was assessed by the rate of reads mapping to the genome with BWA (version 0.717) (13), which found that 99.98% could be aligned.

The genome was also annotated with multiple software. PASA (version 2.3.3) was used for gene prediction with RNA-Seq data (14). *De novo* gene prediction was performed using AUGUSTUS (version 3.3.1), GeneMark-ES (version 4, 2018), and GlimmerHMM (version 3.0.4) (15–17). The prediction was also performed by aligning homologous proteins from *Colletotrichum* spp. to the genome using GeMoMa (version 1.6.1) (18). Gene prediction results were integrated into a unified gene model set by Evidence Modeler (version 1.1.1), which was further refined by TransposonPSI to remove genes derived from transposons (19). The final predicted gene count was 13,129. The assembly and predictions were evaluated using BUSCO (version 5.2.2 and version 3.0.1) with fungi_odb10 (--mode genome --lineage_dataset fungi and -l BUSCO_db -m prot). The statistics of the genome and annotation are shown in Table 1. The complete genome and annotation of *C. jinshuiense* are expected to help to understand the pathogenicity and pathogen-host interaction mechanisms.

**TABLE 1:** Genome statistics and annotation information of *C. jinshuiense* EsH8

Assembly parameters	Value
Sequencing platform	PacBio Reivo and MGI DNBSEQ-T7 PE150
Sequencing and assembly	
No. PacBio reads	503,022
*N*_50_ of the PacBio reads	20.54 kb
Total no. of PacBio bases	10,041,549,414
Genome coverage of PacBio bases	192.37
No. MGI reads	34,138,689
Genome coverage of MGI bases	94.71
Assembly level	Complete
Genome size	52.13 Mb
Sequencing coverage	287×
Number of contigs/scaffold	15
Average contig/scaffold	3,258,231
Contig *N*_50_	5,052,393
Maximum chromosome	6,212,716
GC content	52.70%
Assembly BUSCO evaluation (%)	
Complete	98.42
Single copy	97.76
Duplicated	0.66
Fragmented	0.53
Missing	1.06
Fungi	100.00
Genome annotation	
PASA	7,476
Augustus	12,232
GeneMark-ES	11,925
GlimmerHMM	13,153
GeMoMa	14,051
Evidence Modeler	13,210
TransposonPSI	81
Finalset	13,129
Prediction BUSCO evaluation (%)	
Complete	99.74
Single copy	99.34
Duplicated	0.40
Fragmented	0.00
Missing	0.26
Fungi	100.00

## Data Availability

The complete genome sequence of *C. jinshuiense* isolate EsH8 has been deposited in GenBank under accession (CP150798-CP150813). The raw sequence reads were deposited in the Sequence Read Archive (SRA) under accession numbers SRR28087747 (PacBio), SRR28087748 (MGI), and SRR28087746 (RNA-Seq). The associated BioProject and BioSample accession numbers are PRJNA1080248 and SAMN40122905, respectively.
